# Hepatic and serum lipid signatures specific to nonalcoholic steatohepatitis in murine models

**DOI:** 10.1038/srep31587

**Published:** 2016-08-11

**Authors:** Franck Chiappini, Christophe Desterke, Justine Bertrand-Michel, Catherine Guettier, François Le Naour

**Affiliations:** 1 Inserm, Unité 1193, Villejuif, F-94800, France; 2 Univ Paris-Sud, Institut André Lwoff, Villejuif, F-94800, France; 3 DHU Hepatinov, Villejuif, F-94800, France; 4 Inserm, US33, Villejuif, F-94800, France; 5 MetaToul-Lipidomic Facility, MetaboHUB, Inserm UMR1048, Toulouse, F-31432, France; 6 AP-HP Hôpital du Kremlin-Bicêtre, Service d’Anatomopathologie, Le Kremlin, F-94275, France

## Abstract

Nonalcoholic fatty liver (NAFL) is a precursor of nonalcoholic steatohepatitis (NASH), a condition that may progress to cirrhosis and hepatocellular carcinoma. Markers for diagnosis of NASH are still lacking. We have investigated lipid markers using mouse models that developed NAFL when fed with high fat diet (HFD) or NASH when fed using methionine choline deficient diet (MCDD). We have performed a comprehensive lipidomic analysis on liver tissues as well as on sera from mice fed HFD (n = 5), MCDD (n = 5) or normal diet as controls (n = 10). Machine learning approach based on prediction analysis of microarrays followed by random forests allowed identifying 21 lipids out of 149 in the liver and 14 lipids out of 155 in the serum discriminating mice fed MCDD from HFD or controls. In conclusion, the global approach implemented allowed characterizing lipid signatures specific to NASH in both liver and serum from animal models. This opens new avenue for investigating early and non-invasive lipid markers for diagnosis of NASH in human.

Nonalcoholic fatty liver disease (NAFLD) is a pathological condition exhibiting a wide spectrum of lesions from nonalcoholic fatty liver (NAFL), to nonalcoholic steatohepatitis (NASH). It is established that NASH may progress to hepatic fibrosis, cirrhosis and hepatocellular carcinoma^1^,^2^,^3^. NAFLD is a systemic disease associated with obesity, insulin resistance, type 2 diabetes mellitus and metabolic syndrome^4^,^5^,^6^,^7^. The dramatic increase in such incidences that currently more than 1 billion individual, makes NAFLD the most common cause of chronic liver diseases and a major public health problem worldwide^8^,^9^,^10^,^11^.

The hallmark of fatty liver disease is the intra-cellular accumulation of lipids, resulting in the formation of lipid droplets into hepatocytes. This accumulation results from an imbalance between uptake, synthesis, export and oxidation of fatty acids^4^,^12^,^13^,^14^,^15^,^16^,^17^,^18^,^19^,^20^,^21^,^22^. Since not all lipids are created equal, lately in the search for markers of NASH, comprehensive lipidomic studies were performed from liver biopsies or sera using human samples or mouse models^12^,^23^,^24^,^25^,^26^,^27^. These studies revealed alterations in homeostasis of some lipids during the progression of NASH. However, none of these studies was able to characterize a specific lipid signature of NASH due to the lack of appropriate statistical procedures.

In this study, we used well-established dietary mouse models of NAFL and NASH. We have implemented an approach based on comprehensive lipidomic analysis followed by learning-machine data analysis and confirmed these results by an unsupervised clustering analysis. This original data analysis has identified lipid signatures in both liver and serum specific to NASH.

## Results

### Characterization of animal models of nonalcoholic fatty liver and steatohepatitis

**Figure 1:**
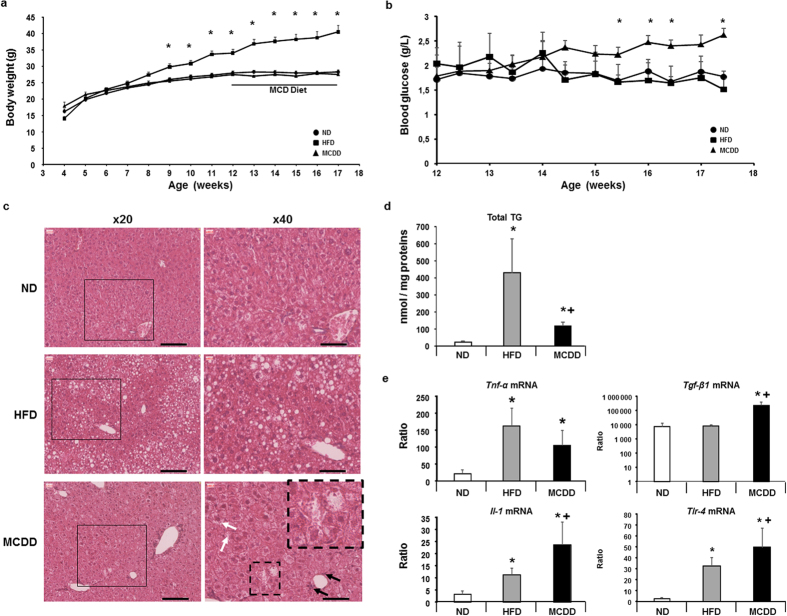
Characteristics of mouse models fed a high-fat diet and a methionine-choline deficient diet. (**a**) Body weights of mice fed a high-fat diet 60% (HFD, ■), a methionine choline deficient diet (MCDD, ▲) and their respective control groups fed a normal diet (ND, •). Mice on MCDD were fed during 5 weeks (black bare). (**b**) Blood glucose follow-up during the 5 weeks when mice were fed a MCDD (▲) compared to HFD (■) and their respective control groups fed a ND (•). (**c**) Hematoxylin and eosin staining of livers from mice fed a HFD, a MCDD and their respective controls (ND). Left panels are 20x magnification and black square area of 40x magnification on the right panels. Scale bares: 100 μm. Black arrows show inflammatory cells and black dash square focus on hepatocytes presenting ballooning and Malory’s body (magnification of black dash square focus on upper-right panel) with presence of hepatic lipid droplets characteristic of NASH. (microvesicular steatosis; white arrows). (**d**) Total hepatic triglycerides (TG) in mice fed a HFD and a MCDD compared to their respective control (ND). (**e**) Hepatic mRNA gene expression levels involved in the inflammatory process and partially controlled by lipids. Data are means ± SEM. *p < 0.05, by unpaired *t*-test compared to mice fed a ND and +p < 0.05, by unpaired *t*-test compared to mice fed a HFD, after ANOVA-test. ND n = 10, HFD n = 5, MCDD n = 5. Il: interleukin; Tgf: transforming grouth factor; Tlr: toll-like receptor; Tnf: tumor necrosis factor.

NAFL and NASH can be induced in mice by using specific diets such as high-fat diet (HFD) and methionine choline deficient diet (MCDD) two well-known models of NAFLD respectively^15^,^28^,^29^. After weaning, mice fed with HFD (n = 5) for 13 weeks showed significant increase in body weight starting after 9 weeks with no difference in blood glucose in fed state compared to their respective control (n = 10) fed with normal diet (Fig. 1a,b). Histological analysis of the liver tissue from mice fed a HFD showed fatty liver, mostly macrovacuolar steatosis around portal tract, associated to significant increase in total triglycerides (Fig. 1c). On the other hand, mice fed with MCDD for 5 weeks had normal body weight but a significant increase in blood glucose in fed state (starting after 3 weeks feeding with MCDD) compared to mice fed a HFD or normal diet (Fig. 1a,b). Histological analysis of the liver tissue from MCDD mice showed macrovacuolar and microvesicular steatosis (Fig. 1c) associated to a significant but moderate accumulation in total triglycerides (Fig. 1d) compared to HFD mice (Fig. 1e). Mice fed HFD developed steatosis without any histological sign of inflammation whereas mice fed a MCDD developed less pronounced steatosis but exhibited ballooning hepatocytes, Mallory hyaline bodies and inflammatory infiltrates (Fig. 1c, lower right panel) as shown before^30^,^31^,^32^.

Inflammation was also addressed at the molecular level by investigating the hepatic mRNA expression level of genes implicated in the inflammatory process such as *Tnf-α*, *Tlr-4, Il-1* and *Tgf- β1*^33^. These genes were significantly increased in liver of mice fed a MCDD (Fig. 1e).

Importantly, because of the short period on MCDD none of these mice exhibited fibrosis placing the study at an early step during NASH progression avoiding then any metabolic consequences on hepatocytes.

### Hepatic lipid signature of nonalcoholic steatohepatitis

**Figure 2:**
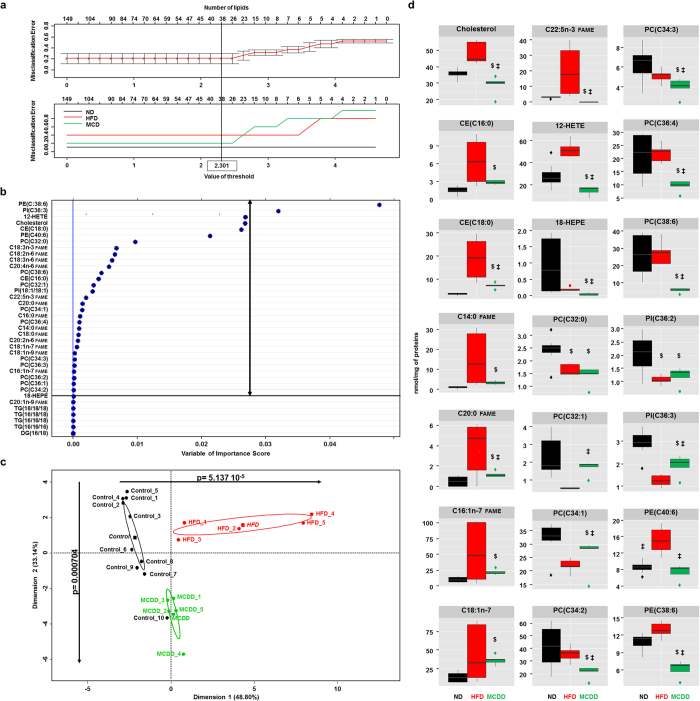
Specific hepatic lipids discriminating mice fed a MCDD. (**a**) Identification of 38 lipids among 149 lipids based on the calculating threshold 2.301 to have the minimum misclassification rate between the 3 groups of mice using the algorithm based on prediction analysis of microarray. (**b**) 32 lipids identified among 38 lipids discriminating the three groups of mice based on random forests analysis. (**c**) Principal component analysis (PCA) using 32 lipids identified in (A). Dots represent each mouse, lines are the ellipses centred to the mean (coloured squares) representing 95% interval confidence, and *p* the probability associated with the F- test of the analysis of variance along the axes of the first and the second dimensions (α = 0.05). (**d**) Boxplots of the 21 lipids from PCA discriminating mice fed a MCDD. ^$^p < 0.05 compared to mice fed ND; ‡p < 0.05 compared to mice fed HFD based on ANOVA-test follow by unpaired *t*-test. 12-HETE: 12-hydroxy-5Z,8Z,10E,14Z-eicosatetraenoic acid; 18-HEPE: 18-hydroxy-5Z,8Z,11Z,14Z,16E-eicosapentaenoic acid; CE: cholesteryl ester; DG: diacylglycerol; HFD: high-fat diet (red); FAME: fatty acyl methyl ester; MCDD: methionine-choline deficient diet (green); ND: normal diet (black). PC: phosphatidylcholine; PE: phosphatidylethanolamine; PI: phosphatidylinositol; TG: triglycerides. Most representative phospholipids: PC(C32:0) = PC(16:0/16:0); PC(C32:1) = PC(16:0/16:1); PC(C34:1) = PC(16:0/18:1); PC(C34:2) = PC(16:0/18:2); PC(C34:3) = PC(16:1/18:1); PC(C36:4) = PC(18:2/18:12); PC(C38:6) = PC(18:2/20:4); PE(C38:6) = PE(18:1/20:5); PE(C40:6) = PE(18:0/22:6); PI(C36:2) = PI(18:1/18:1); PI(C36:3) = PI(18:1/18:2).

In order to identify lipids discriminating the pathophysiological status of the liver, a comprehensive lipidomic analysis was performed on 20 liver biopsies from animals fed a HFD (n = 5) or a MCDD (n = 5) and their respective controls (n = 10) fed with normal diet. Lipids were extracted from the liver tissue and further identified and quantified by gas phase or liquid phase chromatography coupled to mass spectrometry. Investigations were conducted on 149 lipid species such as cholesterol and cholesteryl esters (CE), diglycerides (DG), triglycerides (TG), free fatty acids (FFA), fatty acyl methyl ester (FAME), eicosanoids and phospholipids (see Supplementary Fig. S1a,b) associated to a significant increase in ω-6 to ω-3 ratio in the liver of both mice fed a HFD and MCDD whereas the ω-3 index was significantly decreased only in mice fed a MCDD (see Supplementary Fig. S1c), two indexes that are surrogates of pro-inflammatory state^34^,^35^,^36^. We first focused on the identification of the lipids discriminating the three groups of mice. We first performed a statistical approach called prediction analysis of microarrays (PAM) that allowed calculating the threshold value giving the minimum cross-validated misclassification error rate^37^. Thus, the threshold value was 2.301 leading to identify 38 lipids out of 149 for distinguishing control (*i.e.* normal diet), HFD and MCDD mice with a level of global misclassification estimated to 23.3% (Fig. 2a and see Supplementary Fig. S2a–c). The statistical analysis was further pursued by using random forests on the set of 38 lipids. Indeed, random forests is a recent mathematical method that allows comparing different groups exhibiting high number of variables and then defining those variables which are predictors of a given status^38^. Thus, random forests analysis led to refine from 38 to 32 the number of variables (Fig. 2b) separating the three groups of mice as visualized by plotting on a two-dimensional graph using principal component analysis (Fig. 2c). Then, the principal component analysis allowed selecting 21 lipids with high significance (Fig. 2c) thus constituting the minimal signature discriminating mice fed a MCDD. The hepatic lipid signature of NASH was constituted by cholesterol and 2 cholesterol esters, 7 fatty acids and 11 phospholipids (Fig. 2d). Then, this statistical approach was validated by using unsupervised clustering DIANA (dendrogram with Euclidean distance) based on the 21 lipids identified. Indeed, the heat map showed that the groups of mice including mice fed a MCDD were well clustered (see Supplementary Fig. S2d).

Altogether, these results demonstrated that comprehensive lipidomic analysis combined to data learning approaches succeeded to characterize a specific hepatic lipid signature of the grades of fatty liver diseases, in particular of NASH.

### Serum lipid signature of nonalcoholic steatohepatitis

**Figure 3:**
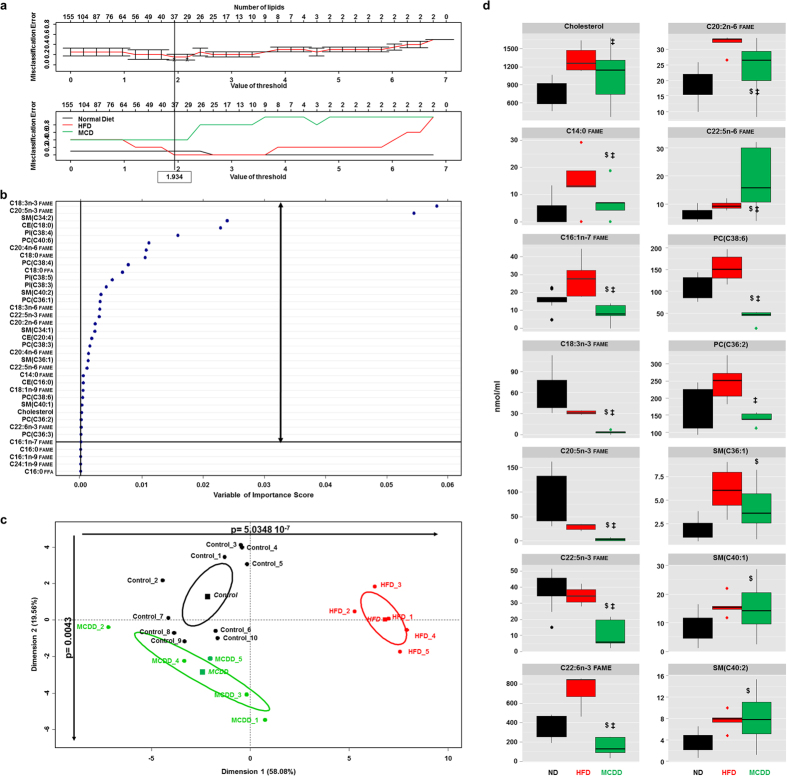
Specific serum lipids discriminating mice fed a MCDD. (**a**) Identification of 37 lipids among 155 lipids based of the calculating threshold 1.934 to have the minimum misclassification rate between the 3 groups of mice using prediction analysis of microarray. (**b**) 33 lipids identified among 37 lipids discriminating the three groups of mice based on random forest analysis. (**c**) Principal component analysis (PCA) using 33 lipids identified in (A). Dots represent each mouse, lines are the ellipses centred to the mean (coloured squares) representing 95% interval confidence, and *p* the probability associated with the F- test of the analysis of variance along the axes of the first and the second dimensions (α = 0.05). (**d**) Boxplots of the 14 lipids from PCA discriminating mice fed MCDD. ^$^p < 0.05 compared to mice fed ND; ^‡^p < 0.05 compared to mice fed HFD based on ANOVA test follow by Student *t*-test. CE: cholesteryl ester; HFD: high-fat diet (red); FAME: fatty acyl methyl ester; FFA: free fatty acid; MCDD: methionine-choline deficient diet (green); ND: normal diet (black); PC: phosphatidylcholine; PI: phosphatidylinositol; SM: sphingomyelin. Most representative phospholipids: PC(C38:6) = PC(18:0/20:6); PC(C36:2) = PC(18:1/18:1); SM(C36:1) = SM(18:1/18:0); SM(C40:1): SM(18:1/22:0); SM(C40:2): SM(18:1/22:1).

A specific lipid signature of NASH in the serum was we further investigated from the same series of animals described previously. Comprehensive lipidomic analysis identified and quantified a total of 155 lipid species in such sera (see Supplementary Fig. S1d and e). We also found in the serum a significant increase in ω-6 to ω-3 ratio and a significantly decreased whereas the ω-3 index in serum of mice fed a MCDD (see Supplementary Fig. S1f), Identification of the lipids discriminating the three groups of mice was performed using the same statistical approach PAM that has calculated the threshold value at 1.934 leading to identify 37 lipids out of 155 for distinguishing control (*i.e.* normal diet), HFD and MCDD mice with a level of global misclassification estimated to 16.6% (Fig. 3a and Supplementary Fig. S3a–c). The statistical analysis was further pursued by using random forests on the set of 37 lipids leading to refine from 37 to 33 the number of variables separating the three groups of mice as visualized by plotting on a two-dimensional graph using principal component analysis (Fig. 3b,c). Then, the principal component analysis allowed selecting 14 lipids with high significance (Fig. 3c) thus constituting the minimal signature discriminating mice fed MCDD. The serum lipid signature of NASH was constituted by cholesterol, 8 fatty acids and 5 phospholipids (Fig. 3d). Thus the heat map obtained again by using unsupervised clustering DIANA based on the 14 lipids identified, showed that all groups including mice fed a MCDD were well clustered (see Supplementary Fig. S3d).

Altogether, these results allowed characterizing a specific serum lipid signature of the grades of fatty liver diseases, in particular mice fed MCDD which developed NASH.

### Comparison of hepatic and serum signatures of nonalcoholic steatohepatitis

The comparison between both hepatic and serum lipid signatures of NASH was investigated (see Supplementary Fig S1). We first compared the whole lipid composition from liver tissues and sera that led to the identification of 149 and 155 lipids in the three groups of mice, respectively. No difference was observed in the distribution in each lipid family (χ^2^ = 0.242; see Supplementary Fig. S1a,c). The rates of saturated fatty acids (SFA), monounsaturated fatty acids (MUFA) and polyunsaturated fatty acids (PUFA) of FFA group and of FAME group detected in the liver were also not different (χ^2^ = 0.670; see Supplementary Fig. S1b), as well as in the serum (χ^2^ = 0.845; see Supplementary Fig. S1d). Furthermore, the comparison of SFA, MUFA and PUFA rates of FFA and FAME between liver and serum showed again no difference (χ^2^ = 0.979 and χ^2^ = 0.887, respectively; see Supplementary Fig. S1b,d). These results indicated that there was no discrepancy in overall lipid composition between liver and serum. Then, the study was further focused on the comparison of the 21 lipids found in the liver and the 14 lipids found in the serum of mice fed a MCDD. Only four lipids appeared as common between liver and serum namely cholesterol, myristic acid (C14:0), palmitoleic acid (C16:1n-7) and docosapentanoic acid (C22:5n-3). These observations suggested that the hepatic and serum lipid signatures of NASH have to be considered as mostly independent.

## Discussion

This study highlighted the major interest of combining a global approach as lipidomic with bioinformatic specific strategy such as PAM followed by an unbiased learning machine statistical approaches (*i.e.* random forests)^38^,^39^,^40^,^41^. Such statistical workflow allowed identifying a hepatic signature of 21 lipids and a serum signature of 14 lipids specific of MCDD fed mice and was validated by using an unsupervised clustering analysis.

Lately, MCDD model of NASH was the best dietary model to mimic rapidly the human pathology without any direct influence of the diet and/or genetic background that can modify subsequently the hepatic and serum lipid composition^15^,^28^,^42^,^43^. Then, the statistical workflow approach was able to identify hepatic and serum lipids that were already found significantly increased or decreased in patients or mouse models of NAHS^14^,^23^,^24^,^44^,^45^,^46^,^47^,^48^,^49^,^50^,^51^,^52^,^53^, but none of these lipids were identified as biomarkers in these studies until now.

Actually, the whole lipid combination constituting each signature in the liver or in the serum is the key to discriminate the NASH group, rather than considering each lipids alone.

Lipids from both signatures such as fatty acids, phospholipids, cholesterol, sphingomyelins and eicosanoids (*e.g.* 12-HETE, 18-HEPE) are known to play a role in the structure of membranes or in cell signalling^12^,^16^,^21^,^54^. Thus, the signatures may reflect major impact of liver metabolic diseases on cellular processes. The lipids from the signatures may also trigger inflammation^17^,^24^,^48^,^55^,^56^,^57^,^58^,^59^,^60^,^61^. Interestingly, lipid signatures correlated with molecular markers of inflammation in our murine models. Increasing evidence suggest that the modulation of lipid-signalling using TLR-4 pathway for activating macrophages represent (*e.g.* release of TNF-α) a common pathogenic mechanism underlying lipotoxicity in NASH^62^,^63^. Fatty acids promote TNF-α-induced hepatocyte cell death and the activation of immune system^64^,^65^,^66^,^67^,^68^. Release of TNF-α also induced production of other pro-inflammatory cytokines according to our observations (*e.g*. IL-1, TGF-β1). Histological examinations of liver tissue of mice fed a MCDD after only 5 weeks exhibited early signs of NASH with an inflammation and no fibrosis, suggesting that the metabolic disorders and thus the resulting lipid signatures may constitute an early event during the progression to NASH.

In conclusion, we clearly developed a new data mining workflow approach to characterize specific lipid signatures at early stage of NASH in both liver and serum. This new approach has to be now applied in human clinical trials. This opens new avenues for non-invasive early diagnosis of NASH and follow-up of patients with NAFLD.

## Methods

### Animal Models

Male C57Bl/6J mice were fed on normal diet (ND), high-fat diet (HFD) and methionine-choline deficient diet (MCDD). A total of 20 animals underwent ND (n = 10, Teklad Rodent Diet no. 5053; 5% kcal from fat; 3.1 kcal/g), HFD (n = 5; 13 weeks on diet, Research Diet D12492i; 60% kcal from fat; 5.24 kcal/g) and MCDD (n = 5; 5 weeks on diet, Teklad, Ref# TD.90262). Mice fed HFD and MCDD developed NAFL and NASH, respectively^69^,^70^. Mice were housed at room temperature (22–24 °C) with a 12-hour light/12-hour dark cycle. Food and water were provided *ad libitum*. Body weight of each mouse was recorded every week. The optimal number of animal per study group (n = 5) was calculated using *t-*test with 0.05 α error-set, 0.9 for the power (1-β) and based on lipidomic data already published^24^. Then, based on our lipidomic data using total TG in the liver assessed in the three groups of mice, the calculated power was 0.996 using ANOVA one-way post-hoc analysis (GPower software; version 3.1.9.2). All experimental protocols were approved by the Institutional Animal Care and Use Committee (Jackson Laboratories, Main, USA) and by the “Comité National de Réflexion Ethique sur l′Expérimentation Animale 05” (Protocol # Ce5/2012/075, Paris, France). All experiments were performed in accordance with the relevant guidelines and regulation of each country.

### Blood tests

Fed mice were bled between 8 and 10 am. Serum was separated from blood and kept at −80 °C until lipidomic analysis. Blood glucose was assessed by One Touch Ultra Glucometer (Lifescan Inc.) every morning between 8AM and 9AM during the 5 weeks three times a week *via* the tail vein.

### Quantitative Real Time RT-PCR of Genes involved in Inflammatory Process

Total RNA was extracted from liver frozen tissue using RNA-STAT 60 reagent (AMS Biotecnology Europe LTD, UK) and levels were quantified with NanoDrop-ND1000 (Thermo Scientific). cDNAs were generated by using the RivertAid® First Strand cDNA Synthesis (Thermo Scientific, France), and Syber Green from FastStart Essential DNA Green Master mixes (Roche, Life Science) were used to quantify *Tnf-α, Il1-α, Tgf-β1 and Tlr4* mRNA levels with specific primers of each gene described previously^71^ and summarized in Supplementary Table S1.

Quantitative real time PCR was performed by using LightCycler® 96 Instrument (Roche, Life Science) machine. Gene expression levels were normalized to actin RNA levels and data analyzed with LightCycler® 96 SW 1.1 software (Roche, Life Science). For each sample, the gene to *Gadph* ratio was calculated based on an arbitrary value of copies determined by the standard curve for each gene, as described before^72^.

### Histological liver assessments

Livers were removed and fixed in 4% paraformaldehyde in PBS (Sigma-Aldrich) overnight and then embedded in paraffin. Serial 5-μm thick sections were cut on sliding microtome (Microm HM335E, Microm Microtech) and stained either hematoxylin and eosin or picrosirius red by standard procedures. Histological liver assessments were done by pathologist expert based on the definition of NASH^30^,^31^,^32^ and finally the slides were photographed using a model of Scanscope 6 slides (Aperio CS, USA), imaging and analysed by ColaPix software v3.4.3 (TRIBVN, Fr) as used before^72^.

### Lipid Profiling

Liver biopsies were homogenized in 2 ml of methanol/EGTA (2:1 v/v) and in 0.9 ml of HBSS for eicosanoids analysis with FAST-PREP (MP Biochemicals) tissue lyser for further lipid analyses. Also, the equivalent of 0.5 mg of tissues was evaporated. The dry pellets were dissolved in 0.25 ml of NaOH (0.1 M) overnight and proteins were measured with the Bio-Rad assay. The quantification of the lipids is expressed in nmol/mg of total proteins.

Briefly, lipids were extracted from liver tissues (equivalent of 1 mg of issues) or serum (10 μl) according to Bligh and Dyer^73^ in dichloromethane/methanol/water (2.5:2.5:2.1, v/v/v), in the presence of the internal standards (stigmasterol, cholesteryl heptadecanoate, glyceryl trinonadecanoate) to quantify neutral lipids. Dichloromethane phase were evaporated to dryness, and the residue dissolved in 20 μl of ethyl acetate. 1 μl of the lipid extract was analyzed by gas-liquid chromatography on a FOCUS Thermo Electron system using a Zebron-1 Phenomenex fused silica capillary columns according to previous publication^74^. Oven temperature was programmed from 200 °C to 350 °C at a rate of 5 °C per min and the carrier gas was hydrogen (0.5 bar). The injector and the detector were at 315 °C and 345 °C respectively.

Phospholipids for relative quantification were extracted as neutral lipids but with 2% acetic acid in the presence of the internals standards (Cer(d18:1/15:0) 16 ng; PE(12:0/12:0) 180 ng; PC(13:0/13:0) 16 ng; SM(d18:1/12:0) 16 ng; PI(16:0/17:0) 30 ng; PS(12:0/12:0) 156.25 ng). After centrifugation the organic phase was collected and dried under azote, then dissolved in 50 μL of methanol. Sample solutions were analysed using an Agilent 1290 UPLC system coupled to a G6460 triple quadripole spectrometer (Agilent Technologies) and using MassHunter software (Agilent Technologies) for data acquisition and analysis. An Kinetex HILIC column (Phenomenex, 50 × 4, 6 mm, 2, 6 μm) was used for LC separations. The column temperature was controlled at 40 °C. The mobile phase A was Acetonitrile; and B was 10 mM ammonium formate in water at pH 3,2. The gradient was as follows: from 10% to 30% B in 10 min; 10–12 min, 100% B; and then back to 10% B at 13 min for 1 min re-equilibrium prior to the next injection. The flow rate of mobile phase was 0, 3 mL/min and the injection volume was 5 μL. An electrospray source was employed in positive (for Cer, PE, PC and SM analysis) and negative ion mode (for PI and PS analysis). The collision gas was azote. Needle voltage was set at +4000 V.

Several scan modes were used. First, to obtain the naturally different specie’s mass, we analyzed cells lipid extracts with a precursor ion scan of 184 m/z, 241 m/z and 264 m/z to PC/SM, PI and Cer respectively; and a neutral loss scan of 141 and 87 to PE and PS respectively. The collision energy optimums for Cer, PE, PC, SM, PI, PS were 2 eV, 2 eV, 30 eV, 25 eV, 45 eV, and 22 eV respectively. Then the corresponding SRM transitions were used in order to quantify different PL species for each class. Data were treated using QqQ Quantitative (version B.05.00) and Qualitative analysis software (version B.04.00).

For Fatty Acid Methyl Ester (FAME) analysis, liver tissues (the equivalent of 1 mg) or serum (10 μl) were extracted as neutral lipids in the presence of the internal standards glyceryl triheptadecanoate (2 μg) and transmethylated 1 h in boron trifluoride methanol solution 10% at 55 °C. After addition of water (1 ml) to the crude, FAMEs were extracted with hexane (3 ml), evaporated to dryness and dissolved in ethyl acetate (20 μl). FAME (1 μl) was analysed by gas-liquid chromatography on a Clarus 600 Perkin Elmer system using Famewax RESTEK fused silica capillary columns (30 m × 0.32 mm i.d, 0.25 μm film thickness)^75^. Oven temperature was programmed from 110 °C to 220 °C at a rate of 2 °C per min and the carrier gas was hydrogen (0.5 bar). The injector and the detector were at 225 °C and 245 °C respectively.

### Eicosanoid profiling

Around 10 mg of tissue were mixed and homogenized with 300 μL of cold methanol (MeOH) and 5 μL of internal standard (Deuterium labelled compounds). After centrifugation at 900 g for 15 min at 4 °C, supernatants were transferred into 2 mL 96-well deep plates and diluted in 2 mL of pure water. Samples were then submitted to solid phase extraction using HRX 96-well plate (50 mg/well, Macherey Nagel) pre-treated with MeOH (2 mL) and equilibrated with 10% MeOH (2 mL). After sample application, extraction plate was washed with 10% MeOH (2 mL). After drying under vacuum system, lipids were eluted with 2 mL of MeOH. Prior to LC-MS/MS analysis, samples were evaporated under nitrogen gas and reconstituted in 10 μL of MeOH. LC-MS/MS analyses of eicosanoids were performed as previously described^76^. Briefly, lipids were separated on a ZorBAX SB-C18 column (2.1 mm, 50 mm, 1.8 μm; Agilent Technologies) using 1290 Infinity HPLC system (Agilent Technologies) coupled to an ESI-triple quadruple G6460 mass spectrometer (Agilent Technologies). Data were acquired in multiple reaction monitoring mode with optimized conditions (ion optics and collision energy). Peak detection, integration and quantitative analysis were done using Mass Hunter Quantitative analysis software (Agilent Technologies) based on calibration lines built with commercially available eicosanoids standards (Cayman Chemicals)^76^.

### Statistical Analysis

All calculations are performed using R v.3.2.2^77^. To avoid any bias due to animal experiment procedure, prediction analysis for microarrays was used on lipids identified from liver and serum between the four groups of mice define as a control group fed normal diet for mice fed HFD (n = 5), mice fed HFD (n = 5), a control group fed normal diet for mice fed MCDD (n = 5) and mice fed MCDD (n = 5) and including heterogeneity analysis, accurate and fast classifier for discriminating the diseased group mice fed MCDD to the others using “*pamr*” package^78^. The maximum of misclassification error was set-up at 20% for both liver and serum analysis to determine the best threshold for the minimum of lipids discriminating the three groups of mice.

Then, in order to identify the specific lipids that contributed to the significant overall effect between the different groups of mice, random forests was used with “*randomForest*” and “*varSelRF*” packages leading to obtain a narrow numbers of markers^38^,^40^. Briefly, random forests consists of a collection of tree predictors where each tree depended on the value of a random vector of measured variables sampled independently and with the same distribution for all trees in the forest. Random forests classified a case by assigning the input vector of variables to each tree of the forest. Each tree gave a classification, i.e. a classis voted, and the forest chose the class with the most votes from all the trees in the forest^38^,^40^. Random forests analysis was an effective tool in prediction without over-fitting and they have already been used for multiclass classification in gene expression microarray data and for analysis of genetic association studies^38^,^40^,^41^,^79^. Thus to determine the most discriminant lipids, random forests were applied using “*randomForest*” package in R. To determine the best number of predictors (*mtry*) was used for each split of the tree and *tuneRF()* function was used to determine the lowest *mtry* for to the lowest out-of-bag (OOB) error data that was used to get a running unbiased estimate of the classification error as trees were added to the forest. Also *ntree* (number of trees to be built) was set up at 1490 and 1550 for liver and serum, respectively, corresponding to the number of the columns (variable) of the matrix multiply by ten. Indeed, with small sample sizes or even “small n large p” problems, growing large trees usually does lead to the best performance as demonstrated before^39^. During the analysis, the mean decreased accuracy (MDA) and the mean decreased Giny (MDG) were determined. MDA was determined during the OOB error calculation phase and lipids with a large MDA were more important for classification of the data (data not shown). In addition, MDG that was a measure of how each variable contributes to the homogeneity of the nodes and leaves in the resulting random forests was assessed. Lipids that resulted in nodes with higher purity have a higher MDG. Therefore, data were classified based on their variable of importance score.

Principal component analysis (PCA), 95% confident ellipse centre to the mean and lipids of interest were computed and extracted using “*FactoMinR*” package. The global p-value was calculated using the critical probability associated with the F- test of the analysis of variance along the axes of the first and the second dimensions (α = 0.05).

Heat map using unsupervised clustering DIvisive ANAlysis (DIANA dendrogram) with Euclidean distance was built based on the lipids identified in the liver and the serum using *heatmap.2* function in “*gplots*” and “*cluster*” packages, respectively.

Variable were represented as boxplot and tested with analysis of variance (ANOVA-test, one-way) followed by unpaired *t*-test. To test proportion of different lipids in different groups, chi-square (χ^2^) test with Yate’s correction was used. Type I error-set is 5%, two sided.

To compute graphical representations “*gplots*” and “*RColorBrewer*” packages were used.

## Additional Information

**How to cite this article**: Chiappini, F. *et al.* Hepatic and serum lipid signatures specific to nonalcoholic steatohepatitis in murine models. *Sci. Rep.* **6**, 31587; doi: 10.1038/srep31587 (2016).

## Supplementary Material

Supplementary Information
