# Supplementary material for: Hepatic and serum lipid signatures specific to nonalcoholic steatohepatitis in murine models
Source: Sci Rep. 2016 Aug 11;6:31587. doi: 10.1038/srep31587 (PMC4980672; doi:10.1038/srep31587)
Supplement: Supplementary Information [file srep31587-s1.pdf]

Hepatic and serum lipid signatures specific to nonalcoholic steatohepatitis in murine models

Franck Chiappini, Christophe Desterke, Justine Bertrand-Michel,

Catherine Guettier, François Le Naour

Supplementary information (online)

Supplementary Table S1

Supplementary Figure S1

Supplementary Figure S2

Supplementary Figure S3

Supplementary Table S1. Primers used for quantitative RT-PCR

Genes	Primer Forward 5'-3'	Primer Reverse 5'-3'	TM (°C)
Tnf-α	TCTCATGCACCACCATCAAGGACT	ACCACTCTCCCTTTGCAGAACTCA	60
Il1-α	ACGGCTGAGTTTCAGTGAGACCTT	AGGTGTAAGGTGCTGATCTGGGTT	60
Tgf-β	TAAAGAGGTCACCCGCGTGCTAAT	ACTGCTTCCCGAATGTCTGACGTA	60
Tlr4	AACCAGCTGTATTCCCTCAGCACT	ACTGCTTCTGTTCCTTGACCCACT	60
Gadph	CATGTTCCAGTATGACTCCACTC	GGCCTCACCCCATTTGATGT	58

Primers were selected on previews publication[1] and controlled using Primer3 software and BLASTed (Basic Local Alignment Search Tool) on *https://genome.ucsc.edu*

1 Bai, T., Chen, C. C. & Lau, L. F. Matricellular protein CCN1 activates a proinflammatory genetic program in murine macrophages. *Journal of immunology*. **184**, 3223-3232 (2010).

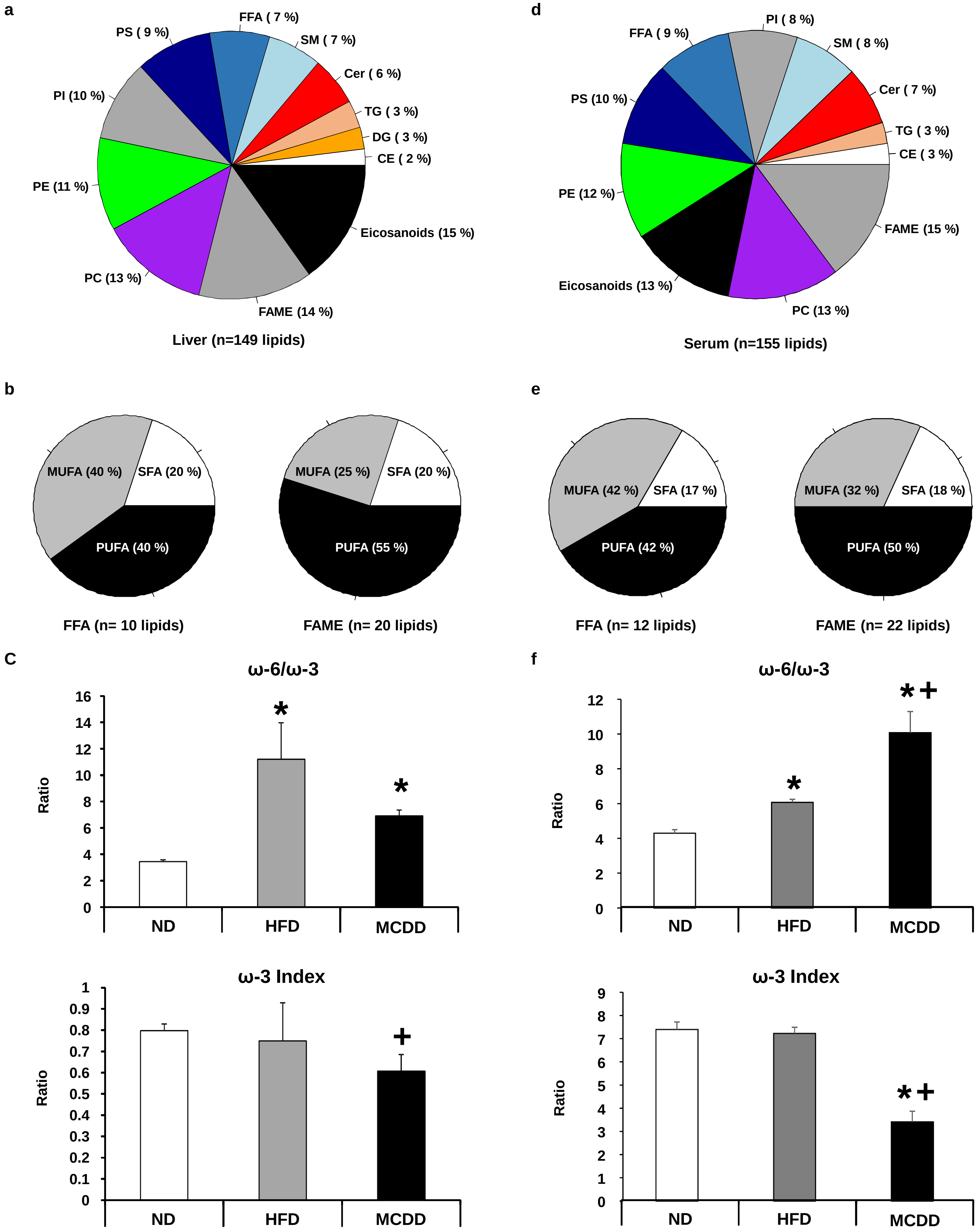

Supplementary Figure S1. No differences in whole-lipid rates between liver and serum. (a) Proportions of lipid family among the 149 lipids in the liver. **(b)** Proportions of SFA, MUFA and PUFA among the 10 FFA and 20 FAME from the 149 lipids in the liver. **(c)** Hepatic ω-6 to ω-3 ratio and ω-3 index (*i.e.* eicosapentaenoic acid and docosahexaenoic acid levels expressed as per cent of total fatty acids) from the different study groups of mice. **(d)** Proportions of lipid family among the 155 lipids in the serum. **(e**) Proportions of SFA, MUFA and PUFA among the 12 FFA and 22 FAME from the 155 lipids in the serum. **(f)** Hepatic ω-6 to ω-3 ratio and ω-3 index from the different study groups of mice. (a, b, d and e) Data are represented by pie diagrams. (c and f) Data are means ± SEM. *p<0.05, by unpaired t-test compared to mice fed a ND after ANOVA test. Normal diet (ND) n= 10; high-fat diet (HFD) n=5; methionine choline deficient diet (MCDD) n=5. CE: cholesteryl ester; Cer: ceramide; DG: diglyceride; FAME: fatty acyl methyl ester; FFA: free fatty acid; MUFA: monosaturated fatty acid; PC: phosphatidylcholine; PE: phosphatidylethanolamine; PI: phosphatidylinositol; PS: phosphatidylserine; PUFA: polyunsaturated fatty acid; SFA: saturated fatty acid; SM: sphingomyelin; TG: triglyceride.

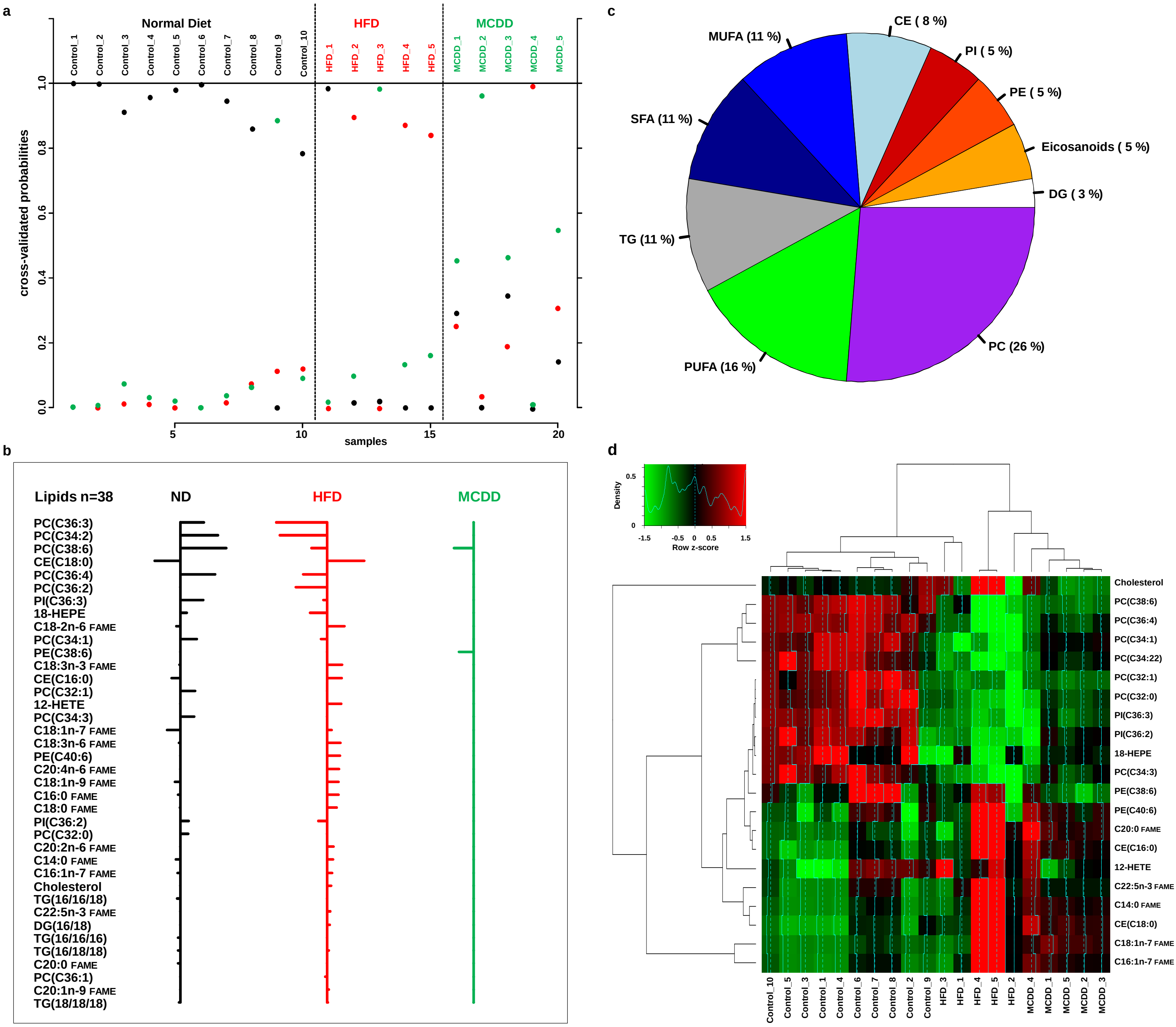

Supplementary Figure S2. Identification of lipids in the liver discriminating the three groups of mice using prediction analysis for microarrays approach. **(a)** Cross-validated probabilities in the three groups of mice based on 2.301 threshold with 23,3% estimated-misclassification error based on the 38 lipids identified. **(b)** Relative abundance of the 38 lipids identified to discriminate each group of mice. **(c)** Lipid family proportion based on the 38 lipids identified. Data are analyzed using prediction analysis for microarrays approach (PAMR package in R) on 148 hepatic lipids identified by mass spectrometry in the three groups of mice (n=10 ND; n=5 HFD, n=5 MCDD). **(d)** Heat map using unsupervised clustering DIvisive ANAlysis (DIANA dendrogram with Euclidean distance) based on 21 lipids identified by random forests analyzed with the 38 lipids identified with PAM. 12-HETE: 12-hydroxy-5Z,8Z,10E,14Z-eicosatetraenoic acid; 18-HEPE: 18-hydroxy-5Z,8Z,11Z,14Z,16E-eicosapentaenoic acid; CE: cholesteryl esters; DG: diglycerides; FAME: fatty acyl methyl ester; HFD: high-fat diet; MCDD: methionine-choline deficient diet; MUFA: monounsaturated fatty acids; ND: normal diet; PC: phosphatidylcholine; PE: phosphatidylethanolamine; PI: phosphatidylinositol; PUFA: polyunsaturated fatty acid; SFA: saturated fatty acid; TG: triglyceride.

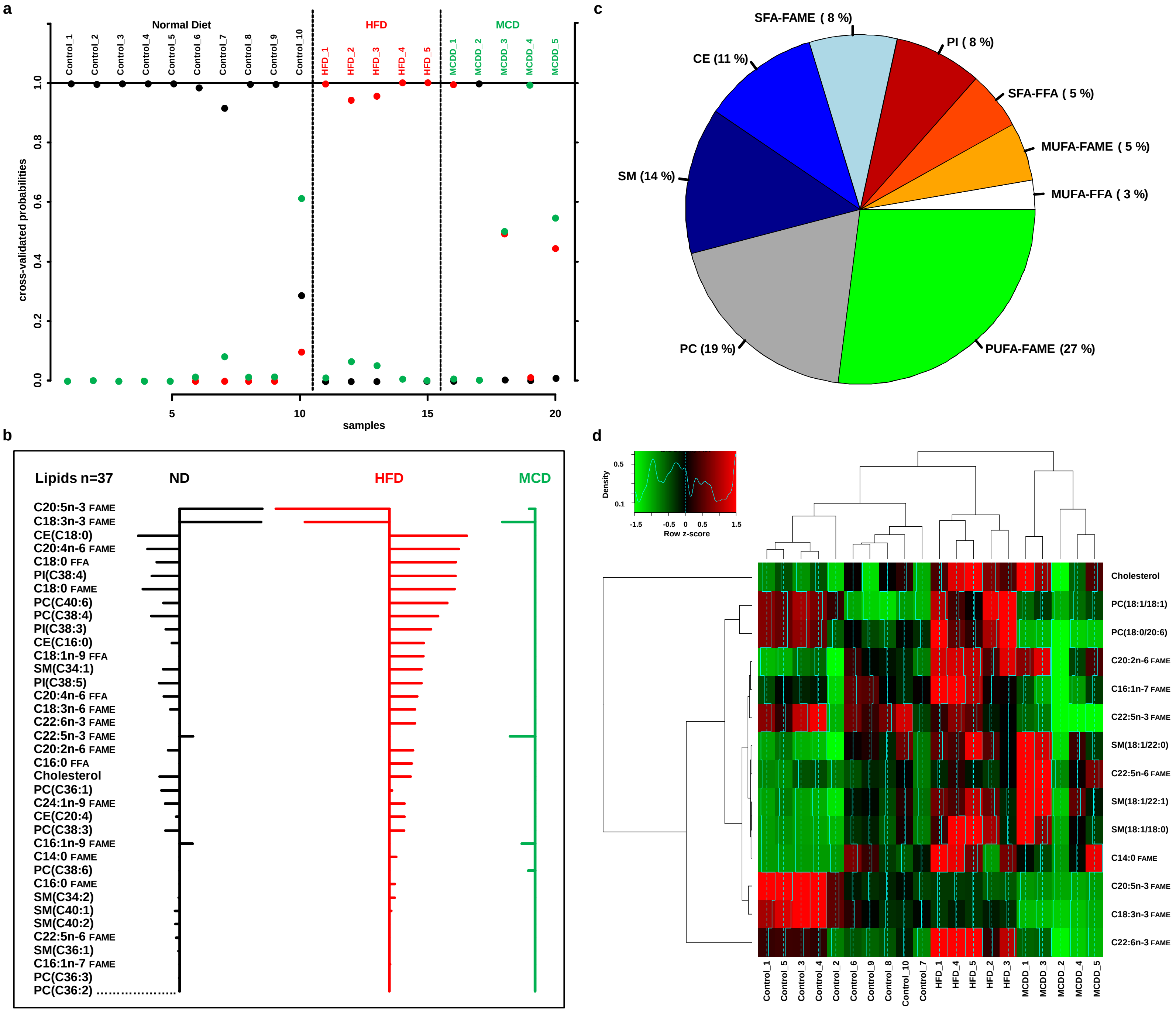

Supplementary Figure S3. Identification of lipids in the serum discriminating the three groups of mice using prediction analysis for microarrays approach. (a) Cross-validated probabilities in the three groups of mice based on 1.934 threshold with 20% estimated-misclassification error based on 37 lipids identified. **(B)** Relative abundance of the 37 lipids identified to discriminate each group of mice. **(C)** Lipid family proportion based on the 37 lipids identified. Data are analyzed using prediction analysis for microarrays approach (PAMR package in R) on 155 hepatic lipids identified by mass spectrometry in the three groups of mice (n=10 ND; n=5 HFD, n=5 MCD). **(d)** Heat map using unsupervised clustering DIvisive ANAlysis (DIANA dendrogram with Euclidean distance) based on 21 lipids identified by random forests analyzed with the 38 lipids identified with PAM. CE: cholesteryl ester; FAME: fatty acyl methyl ester; FFA: free fatty acid; HFD: high-fat diet; MCD: methionine-choline deficient diet; MUFA: monounsaturated fatty acid; ND: normal diet; PC: phosphatidylcholine; PI: phosphatidylinositol; PUFA: polyunsaturated fatty acid; SFA: saturated fatty acid; SM: sphingomyelin.
